# Supplementary material for: The association of EGFR amplification with aberrant exon 20 insertion report using the cobas EGFR Mutation Test v2
Source: PLoS One. 2024 Apr 30;19(4):e0301120. doi: 10.1371/journal.pone.0301120 (PMC11060574; doi:10.1371/journal.pone.0301120)
Supplement: S1 File — (DOCX) [file pone.0301120.s001.docx]

Table S1. Primers used for Sanger sequencing

| **Primer** | **Sequence (5′→3′)** |
| --- | --- |
| *EGFR* exon18F | CAAATGAGCTGGCAAGTGCCGTGTC |
| *EGFR* exon18R | CCAAACACTCAGTGAAACAAAGAG |
| *EGFR* exon19F | GCTGGTAACATCCACCCAGA |
| *EGFR* exon19R | AGCAGGGTCTAGAGCAGAGC |
| *EGFR* exon20F | CCATGAGTACGTATTTTGAAACTC |
| *EGFR* exon20R | CCATGAGTACGTATTTTGAAACTC |
| *EGFR* exon21F | CAGCCATAAGTCCTCGACGTGG |
| *EGFR* exon21R | CATCCTCCCCTGCATGTGTTAAAC |

Abbreviation: *EGFR*, epidermal growth factor receptor

Table S2. Cases with inconsistent ex20ins results by Sanger sequencing and Idylla EGFR assay

|  | cobas test | Sanger sequencing | Idylla EGFR assay |
| --- | --- | --- | --- |
| 1 | ex20ins+ | *p.N771_P772insPGN | WT |
| 2 | ex20ins+ | *p.P772_H773insVGNP | WT |
| 3 | ex20ins+ | *p.P772_H773insGNP | WT |
| 4 | ex20ins+ | *p.N771>DY | WT |
| 5 | ex20ins+ | *p.H773_V774insNPH | WT |
| 6 | ex20ins+ | p.V769_D770insASV | WT |
| 7 | ex20ins+ | *p.N771>GY | WT |
| 8 | ex20ins+ | p.D770_N771insSVD | WT |
| 9 | ex20ins+ | *p.N771>GY | WT |
| 10 | ex20ins+ and ex19del | ex19del | ex20ins, ex19del |

*ex20ins mutation types not included in the cobas and Idylla assays

Abbreviations: ex20ins, exon 20 insertion; ex19del, exon 19 deletion; EGFR, epidermal growth factor receptor; WT, wild type

Table S3. Clinicopathological features of cases (n=104) with different Colorado scores of *EGFR* FISH

| Colorado score | Score 6 | Score 5 | Score 1–4 | *p*-value |
| --- | --- | --- | --- | --- |
| Case number (n) | 46 | 29 | 29 |  |
| Sex (male:female) | 18:28 | 13:16 | 7:22 | 0.233 |
| Mean age (years) | 66.5 | 62.7 | 62.6 | 0.270 |
| Tumor content (%) | 44.5 | 27.6 | 24.3 | <0.001 |
| Histologic grading (%) |  |  |  | <0.001 |
| 1 (lepidic) | 0 | 0 | 3 |  |
| 2 (acinar/papillary) | 0 | 6 | 16 |  |
| 3 (micropapillary, solid, complex glandular structures) | 46 | 23 | 10 |  |
| Necrosis (%) | 27 | 8 | 2 | <0.001 |

Abbreviations: *EGFR*, epidermal growth factor receptor; FISH, fluorescence in situ hybridization
