# Supplementary material for: The association of EGFR amplification with aberrant exon 20 insertion report using the cobas EGFR Mutation Test v2
Source: PLoS One. 2024 Apr 30;19(4):e0301120. doi: 10.1371/journal.pone.0301120 (PMC11060574; doi:10.1371/journal.pone.0301120)
Supplement: Attachment — Submitted filename: Response to Reviewers 1208.docx [file pone.0301120.s003.docx]

Response to Reviewers

Dear editor and reviewers,

We all are grateful for your comments and revised our manuscript according to your suggestions.

**Reviewer #1:** I appreciate the opportunity to provide a peer-review of such an interesting article. The authors present a scenario that is rarely contemplated in our everyday clinical practice, which is to evaluate whether the results of a test to determine EGFR mutations, previously validated by regulatory agencies, could lead to aberrant results. The authors noticed that the introduction of the new technique (Cobas) resulted in a higher frequency of co-occurring mutations (considered very rare), including both typical EGFR mutations and the relatively uncommon ex20ins mutations, which are traditionally considered mutually exclusive. Subsequently, they re-examined the samples using alternative methods, including Sanger, Idylla, and even NGS, only to find that none of these techniques confirmed the presence of ex20ins mutations. Moreover, the authors noted that in cases where Cobas detected ex20ins mutations while Sanger did not, more aggressive disease characteristics were observed, which instigated investigations into EGFR amplification. The finding is interesting as it suggests the possibility that EGFR amplification might be a contributing factor to the aberrant results reported by the Cobas technique. Finally, the authors conclude with the recommendation to validate ex20ins results (whit another test) when reported by the Cobas technique, and that EGFR amplification can lead to aberrant ex20ins mutation results. Given that recent data on the benefit of poziotinib and amivantamab in patients with ex20ins have been reported, this recommendation is particularly relevant. Additionally, given the regulatory agencies' endorsement of the Cobas test, any article that calls its accuracy into question should provide a comprehensive and meticulously structured body of evidence to support the hypotheses of aberrant outcomes. That said, I have some major concerns along with some minor ones:
 
Major Comments:
1. In the third paragraph of the introduction, some procedures performed in the study are described, and results of these studies are also described. This should be specified in detail in the methods and results sections, respectively. I suggest removing this information from the introduction section. On the other hand, within the introduction, I suggest including statements about the Cobas and Sanger techniques since the entire article is about the accuracy of these tests, so it is important to have a paragraph with data about the accuracy of these platforms and what is known about these tests.

**Response to Reviewer:
Thank you for your suggestion. We've added information regarding the accuracy of these tests after the second paragraph of our introduction:**

**「Several molecular testing methods has seen used to detect druggable EGFR mutations. Sanger sequencing has traditionally been considered as the gold standard method but with a low sensitivity which typically requires 20% of mutated alleles [23]. Real-time polymerase chain reaction (RT-PCR)-based tests have been developed and widely used in Taiwan. The cobas EGFR Mutation Test v2 (Roche Molecular Diagnostics, Pleasanton, CA, USA) is an FDA approved RT-PCR test detecting 42 specific mutations in exons 18 to 21 of the human EGFR gene, and it is more sensitive than Sanger sequencing with a detection limit of ~5% of mutant alleles. The IdyllaTM EGFR Mutation Test (Biocartis, Mechelen, Belgium) is a fully automated RT-PCR based molecular diagnostic kit for the qualitative detection of 51 EGFR mutations for FFPE tissue samples with a limit of detection ~2-5% of variant allele frequency [24,25]. Next-generation sequencing (NGS) is more sensitive than RT-PCR based tests with a limit of detection around 1% variant allele frequency [26].」**

2. In the final part of the introduction, the authors state, "... The study aimed to COMPARE the clinical-pathological features and EGFR amplification status ...", however, the study's conclusions indicate that the ex20ins test reported by Cobas should be validated by other techniques and that EGFR amplification can lead to aberrant results. The study's conclusions do not align with the study's objective. I kindly suggest that the authors describe the study's objective in a way that the conclusions correspond to that objective.

**Response to Reviewer:**

**Thank you for your thoughtful consideration of our study and and providing valuable insights about the match between our objective and conclusions. Based on your suggestion, we'll adjust the study's objective in the final part of the introduction to better match our research path and align with the conclusions:**

**「In summary, we reported cases reported to have EGFR ex20ins mutation by the cobas test but unable to be validated by Sanger sequencing, Idylla assay, or liquid biopsy. Comparing with ex20ins cobas+/Sanger+ cases, these ex20ins cobas+/Sanger- cases groups were significantly associated with higher tumor contents, high-grade patterns, tumor necrosis, lower IC Ct value, and EGFR amplification. Based on the low IC Ct values and EGFR FISH results, EGFR amplification may have led to aberrant ex20ins results in the cobas report. It is recommended that ex20ins mutation reported by the cobas test should be validated by other tests (such as the Idylla assay or NGS), particularly in cases with concomitant classic EGFR mut+/ex20ins+.」**

3. Within the methods section, the authors do not provide details about the study's design. Based on the study's features and the data gathered, it appears to be a cross-sectional, descriptive study. I would recommend that they clarify the study's design, because the results are hypothesis generating, and this should be taken into account when analyzing results.

**Response to Reviewer:**

**Thank you for your comment. We added a brief description of our study design in the last part of our instruction:
「This study aimed to validate the ex20ins results reported by the cobas test and to determine whether the different testing sensitivities between cobas and Sanger sequencing leading to the disparity of ex20ins results. Clinicopathological features including tumor contents were compared between cobas+/Sanger+ and cobas+/Sanger- ex20ins groups, and the ex20ins results were validated by Idyalla EGFR Assay. The observation that Sanger sequencing had higher agreement with Idylla and the lower IC Ct value in the cobas+/Sanger- ex20ins group raised the suspicion that high EGFR copy numbers may be associated with aberrant cobas ex20ins report. Fluorescence in situ hybridization (FISH) was used to determine whether there were differences in EGFR gene copy numbers like amplification between these two groups. Finally, medical records including liquid NGS tests and clinical response to EGFR TKIs were reviewed.」**

**We also modified our methods section:
In the first part “Case selection and design of the study”, we added
“This study was a cross-sectional study. The first aim was to compare tumor contents between cobas+/Sanger+ and cobas+/Sanger- ex20ins groups to determine whether the different testing sensitivities of cobas and Sanger sequencing leading to the disparity of ex20ins results. Clinicopathological features including tumor contents were compared between cobas+/Sanger+ and cobas+/Sanger- ex20ins groups. Then Idyalla EGFR Assay was used to validate the ex20ins results of both groups, and the IC Ct values from two groups were compared.”**

**In the second part “Mutation analysis”, we added “IC Ct values between ex20ins cobas+/Sanger+ and cobas+/Sanger- groups.were compared.” after the description of Idylla EGFR Mutation Testing.
In the third part “EGFR FISH”, we added “ FISH was used to compare EGFR gene copy numbers between x20ins cobas+/Sanger+ and cobas+/Sanger- groups using the Colorado Scoring Criteria. Fig 1 shows** the algorithm for groups cases for EGFR FISH..”

4. The article could be structured in a logical sequence that starts with the observation of concurrent mutations => evaluation of ex20ins by other techniques => observation of result discordance => clinical-pathological evaluation and FISH of the samples => Description of aberrant results and the hypothesis that amplification could explain the discordant results. I kindly suggest to the authors to consider restructuring the article in this way
5. I recommend revisiting the title and adjusting the abstract to align with the previous modifications.

**Response to Reviewer:**

**We are grateful for your kind suggestion which makes our manuscript more readable. We rearranged our results according to your recommendation as:
[1] The emergence of cases reported to have concomitant ex20ins and classic EGFR mutations after the use of the cobas test and discrepancies with Sanger sequencing
[2] The cobas+/Sanger- ex20ins group had a higher tumor content, a higher histologic grade, more frequent tumor necrosis, and a lower IC Ct value
[3] Significant disparity in rates of EGFR amplification between cobas+/Sanger- and cobas+/Sanger+ groups
[4] The majority of cases reported having concomitant classic EGFR mut+/ex20ins+ by the cobas test had EGFR amplification
[5] Comparison of cobas testing, Sanger sequencing, EGFR FISH, and liquid NGS results.**

**All authors agreed that our manuscript became more organized after this modification. We all are very grateful for your kind suggestion.**

Minor Comments:
- In the results (Table 01), the high frequency of EGFR mutations found in the reviewed cases (71%) is noteworthy, which could be associated with selection bias, the descriptive design and the retrospective data collection. I kindly suggest to the authors commenting on these findings
- In the third line of the abstract, "ETFT" was written. I suggest correcting this typo
- In Table 01, correct the typo:
- In the "near-loop" row, the last column should have an open parenthesis
- In the "far-loop" row, the last column should have an open parenthesis.
**Response to Reviewer:**

**Thank you so much for your comment. We added 「In addition, the EGFR mutation rate in our department is 71.8% between January 2015 and December 2022, which is higher than the rate reported in previous Taiwanese studies which ranged from 55.5~55.7% [6, 39]. Since our hospital is the largest cancer center in Taiwan and conducting multiple clinical trials of lung cancer, this high EGFR mutation rate may be due to referred cases from other hospitals after diagnosis or disease progression.」in our discussion.**

**We also corrected errors of “ETFT” in our abstract and the Table 1.**

**Reviewer #2:**

1. Please could you elaborate on this statement: "In Taiwan, epidermal growth factor receptor (EGFR) mutations are the most common disease-associated mutations in lung cancer, and several EGFR tyrosine kinase inhibitors (EGFR-TKIs) are used as first-line therapy [4-8]" What does the literature say about the high incidence of lung cancer among Taiwanese people?

**Response to Reviewer:**

**Thank you for raising this point. It has been shown that the EGFR mutation rate is much higher in Asia (~40-50%, including Taiwan, Japan, Korea, and China) than that in the Europe or USA (~15%). According to previous studies in Taiwan, the EGFR mutation rate is around 55%.**

Ref 4: Wu JY, Wu SG, Yang CH, et al. Lung cancer with epidermal growth factor receptor exon 20 mutations is associated with poor gefitinib treatment response. Clin Cancer Res. 2008;14:4877-4882. <https://doi.org/10.1158/1078-0432.ccr-07-5123>
Ref 5: KH Hsu, CC Ho, TC Hsia, et al. Identification of Five Driver Gene Mutations in Patients with Treatment-Naïve Lung Adenocarcinoma in Taiwan. PLoS ONE. 2015;10: e0120852

**We revised our description as:**

**「In Taiwan, epidermal growth factor receptor (EGFR) mutations are the most common disease-associated mutations in lung cancer with an incidence rate around 55% [4, 5], and several EGFR tyrosine kinase inhibitors (EGFR-TKIs) are used as first-line therapy [4-9].」**

2. I would appreciate it if you could provide more details regarding why patients with uncommon EGFR mutations, such as p.G719X, p.S768I, or p.L861Q, typically display limited response to EGFR-TKIs.?

**Response to Reviewer:**

**Thank you for your inquiry regarding the limited response observed in patients with uncommon EGFR mutations, including p.G719X, p.S768I, and p.L861Q, to EGFR tyrosine kinase inhibitors (EGFR-TKIs).**

**Robichaux et al (Nature. 2021 Sep;597(7878):732-737) established the structure-function relationship of EGFR mutations on drug sensitivity and categorized EGFR mutations into four distinct subgroups on the basis of sensitivity and structural changes that retrospectively predict patient outcomes following treatment. These four subgroups are classical-like, T790M-like, ex20 loop insertion, and P-loop αC-helix compression types. Uncommon mutations like G719X, L861Q, and S768I represent mutations proximal to drug binding pocket which has direct or indirect impact on drug binding which make first generation EGFR TKIs less effective.**

**We have added more detailed information in the second paragraph of the article introduction:**

**「****Based on the structural changes and drug sensitivity, EGFR mutations can be separated into four distinct subgroups as classical-like, T790M-like, ex20 loop insertion, and P-loop αC-helix compression types [11]. Uncommon mutations like G719X, L861Q, and S768I represent mutations proximal to drug binding pocket which has direct or indirect impact on drug binding which make first generation EGFR TKIs less effective [8, 11]. Afatinib, a second generation EGFR TKI, has been demonstrated to be more effective for NSCLCs carrying these uncommon mutations and now considered as the first-line therapy with an overall response rate (ORR) exceeding 50% and a median time to treatment failure (TTF) of nearly one year [9, 12].」**

3. The introduction requires significant modifications to imbue a scientific tone.

**Response to Reviewer:
Thank you for your comment. We added a brief description of our study design in the last part of our instruction to delineate our study design and aims.**

**「This study aimed to validate the ex20ins results reported by the cobas test and to determine whether the different testing sensitivities between cobas and Sanger sequencing leading to the disparity of ex20ins results. Clinicopathological features including tumor contents were compared between cobas+/Sanger+ and cobas+/Sanger- ex20ins groups, and the ex20ins results were validated by Idyalla EGFR Assay. The observation that Sanger sequencing had higher agreement with Idylla and the lower IC Ct value in the cobas+/Sanger- ex20ins group raised the suspicion that high EGFR copy numbers may be associated with aberrant cobas ex20ins report. Fluorescence in situ hybridization (FISH) was used to determine whether there were differences in EGFR gene copy numbers like amplification between these two groups. Finally, medical records including liquid NGS tests and clinical response to EGFR TKIs were reviewed.」**

4. The author utilized one-way ANOVA to conduct a comparison between the three groups. I'm interested in knowing the specific criteria considered when choosing Colorado 1 to 4 as the main focus for the analysis. Furthermore, did the author assess the normality of the data prior to conducting this test?

**Response to Reviewer:
Thank you for your comment. The Colorado score has been established and modified by M Varella-Garcia. According to his guidelines for application EGFR FISH to non-small-cell lung and multiple clinical trials, the cut-off value for EGFR FISH positivity was high polysomy (Colorado score 5, defined as ≥4 copies of EGFR in >40% of cells) or EGFR gene amplification (Colorado score=6, defined as (a) an EGFR to CEP7 ratio ≥2 over all scored nuclei and calculated using the sum of EGFR divided by the sum of CEP7 when mean CEP7 per cell is ≥2 copies, or (b) the presence of gene cluster (≥4 spots) in ≥10% of tumor cells, or (c) at least 15 copies of the EGFR signals in ≥10% of tumor cells.). Cases with Colorado score 5 or 6 are considered as EGFR FISH-positive, while those with score 1-4 are considered EGFR FISH-negative according to the guideline [Ref 28]. The background non-tumor cells like inflammatory cells served as normal control for FISH study just like other FISH tests performed in our hospital.**

Ref 28. Varella-Garcia M, Diebold J, Eberhard DA, et al. EGFR fluorescence in situ hybridisation assay: guidelines for application to non-small-cell lung cancer. J Clin Pathol. 2009;62:970-977. doi: 10.1136/jcp.2009.066548.
