# Supplementary material for: The association of EGFR amplification with aberrant exon 20 insertion report using the cobas EGFR Mutation Test v2
Source: PLoS One. 2024 Apr 30;19(4):e0301120. doi: 10.1371/journal.pone.0301120 (PMC11060574; doi:10.1371/journal.pone.0301120)
Supplement: Attachment — Submitted filename: Response to Reviewers 0123.docx [file pone.0301120.s004.docx]

**Response to Reviewers**

Reviewer #1: Dear authors:

I appreciate the willingness and openness to suggestions and comments, as well as your review of the original manuscript. I believe that the findings you wish to convey are particularly relevant to daily clinical practice, as they provide crucial information for decision-making in the management of patients with advanced lung cancer with EGFR mutation. I think you have done a great job in this study. I understand that summarizing and "simplifying" the findings is challenging given the complexity of the results. However, the way you communicate your results is as important as the findings themselves, so presenting them in a scientific and organized manner should be the ultimate goal of the manuscript. Therefore, I still have some significant observations about your manuscript:

**Response to Reviewer:**

**All authors deeply appreciate your review and kind suggestions. We are grateful to have the chance to revise our manuscript. We also uploaded all detailed information in the file “Supplementary information_all cases”.**

* In the introduction section, established knowledge relevant to the study should be included, along with the observations or knowledge gaps that led to conducting the study, without repeating the described information. Therefore, I kindly suggest:

- Consider the possibility of moving the sentence referring to EGFR mutation subgroups that states, “…Based on the structural…” (lines 15 to 18 on page 5) to the end of the first paragraph (after “…afatinib, and osimertinib [4-7]”), and omitting the sentence 'Uncommon mutation like…TKIs less effective [8,11]' (lines 17 to 19, page 5) because it is redundant information already included in the final sentence of the first paragraph. (It would have been helpful to have the draft with line numbers to explain this more effectively).

**Response to Reviewer:**

**Thank you for your kind comment. We moved the sentence “Based on the structural…” to the end of the first paragraph, and omitted the sentence “Uncommon mutation like… TKIs less effective [8,11]” according to your suggestion.**

- On page 6, line 9, from the sentence 'Cell line studies...' to '...resistant to conventional EGFR-TKI...' repeats information already described in line 6 of the same page.

**Response to Reviewer:**

**Thank you for your comment. We deleted “Cell line studies have demonstrated that exon20ins mutations in the near-loop region (positions 767–772) are resistant to conventional EGFR-TKIs but sensitive to poziotinib, while those in the far-loop region (positions 773–774) are sensitive to second-generation EGFR-TKIs (i.e., afatinib) [11]” according to your suggestion.**

- In the penultimate line of page 6, it is stated as a fact that the COBAS method is more sensitive than Sanger. However, since the entire study focuses on the possibility of aberrant findings with the COBAS method, I suggest that the authors consider presenting it not as an established fact but rather as follows: 'Greater sensitivity of the COBAS method has been described.' Similarly, in the paragraph starting on line 10 of page 7.

**Response to Reviewer:**

**We deleted the description of COBAS more sensitive than Sanger and revised the sentence as “The cobas EGFR Mutation Test v2 (Roche Molecular Diagnostics, Pleasanton, CA, USA) is an FDA approved RT-PCR test detecting 42 specific mutations in exons 18 to 21 of the human EGFR gene~~, and it is more sensitive than Sanger sequencing~~ with a detection limit of ~5% of mutant alleles.” We also revised the sentence “Although greater sensitivity of the cobas method has been described, ~~the cobas test is more sensitive than Sanger sequencing,~~ex20ins mutations in many cases (approximately one-third of ex20ins cases reported by cobas) could not be confirmed by Sanger sequencing…” according to your kind suggestion.**

- From line 14 of page 7 ('...Furthermore, cases reported...') to line 5 of page 8, findings from the study are described that should be included in the results section.

**Response to Reviewer:**

**Thank you for your comment. We removed this part from the introduction section.**

- I kindly suggest reviewing the second sentence in the introduction section regarding NCCN recommendations (lines 2 to 5, page 5) and the section about Afatinib (line 19, page 5, to line 1, page 6) to reconsider if the information described there is relevant to the manuscript. My suggestion is to remove them.

**Response to Reviewer:**

**We removed these parts from the introduction section according to your suggestion.**

- In line 16, '… has seen…' is used. I suggest reviewing the typo.

**Response to Reviewer:**

**Thank you for your comment. We replaced “has seen” with “have been”.**

* In the Methods section, I kindly suggest to the authors to organize the included information by first describing the study to be conducted (observational, descriptive, cross-sectional design) based on the observations, and then detailing the selection of cases to be reviewed.

**Response to Reviewer:**

**Thank you for your comment. We added “Based on the observation that cobas test might give aberrant EGFR ex20ins reports, a multicenter, cross-sectional study was designed to compare the ex20ins results among different testing methods” before the detailing the selection of cases to be reviewed according to your kind suggestion.**

* In the Discussion section, the interpretation of the results obtained in the current context and their practical application should be included. The first two paragraphs of the discussion (up to line 12 on page 23) provide information about the EGFR exon 20 insertion mutation and tests for determining EGFR, which should be summarized in the introduction section. I kindly suggest to the authors to review and condense this information together in the introduction, being careful not to repeat the information.

**Response to Reviewer:**

**Thank you for your comment. We summarized and moved the first two paragraphs of the discussion to the introduction section according to your kind suggestion. We also deleted the sentence “The Idylla EGFR assay is a qPCR platform with a limit of detection varying from 2–5% variant allele frequency. It detects 51 EGFR mutations, including exon 18 (p.G719A/S/C), 36 ex19dels, exon 20 (p.T790M, p.S768I), five ex20ins mutations (identical mutation types with the cobas test), and exon 21 point mutations (p.L858R, p.L861Q) [24,25]” in the discussion as this information has been mentioned in the introduction section.**

* I kindly suggest the authors to rewrite the abstract according to the previous changes.

**Response to Reviewer:**

**We are grateful for your kind recommendation and we revised our abstract according to previous changes.**

Best Regards

Reviewer #2: Thank you for your time and effort. The author worked hard to address all the comments I have suggested.

**Response to Reviewer:**

**All authors are grateful for your review and kind suggestions. We are grateful to have the chance to revise our manuscript.**
